# Mindfulness online: a preliminary evaluation of the feasibility of a web-based mindfulness course and the impact on stress

**DOI:** 10.1136/bmjopen-2011-000803

**Published:** 2012-03-05

**Authors:** Adele Krusche, Eva Cyhlarova, Scott King, J Mark G Williams

**Affiliations:** 1 Department of Psychiatry, University of Oxford, Oxford, UK; 2 Mental Health Foundation, London, UK; 3 Wellmind Media Ltd, Brighton, UK

## Abstract

**Objectives:**

Stress has been shown to have a number of negative effects on health over time. Mindfulness interventions have been shown to decrease perceived stress but access to interventions is limited. Therefore, the effectiveness of an online mindfulness course for perceived stress was investigated.

**Design:**

A preliminary evaluation of an online mindfulness course.

**Participants:**

This sample consisted of 100 self-referrals to the online course. The average age of participants was 48 years and 74% were women.

**Interventions:**

The online programme consisted of modules taken from Mindfulness Based Stress Reduction and Mindfulness Based Cognitive Therapy and lasted for approximately 6 weeks.

**Primary and secondary outcome measures:**

Participants completed the Perceived Stress Scale (PSS) before the course, after the course and at 1-month follow-up. Completion of formal (eg, body scan, mindful movement) and informal (eg, mindful meal, noticing) mindfulness activities was self-reported each week.

**Results:**

Participation in the online mindfulness course significantly reduced perceived stress upon completion and remained stable at follow-up. The pre-post effect size was equivalent to levels found in other class-based mindfulness programmes. Furthermore, people who had higher PSS scores before the course reported engaging in significantly more mindfulness practice, which was in turn associated with greater decreases in PSS.

**Conclusions:**

Because perceived stress significantly decreased with such limited exposure to mindfulness, there are implications for the accessibility of mindfulness therapies online. Future research needs to evaluate other health outcomes for which face-to-face mindfulness therapies have been shown to help, such as anxiety and depressive symptoms.

## Article summary

**Article focus:**

- Stress maintained over time can cause a number of negative effects, both physically and psychologically.
- Mindfulness interventions have been shown to have significant beneficial effects to health including significantly decreasing stress.
- Research question: is an online mindfulness course a feasible way to provide an intervention and decrease perceived stress?

**Key messages:**

- An online mindfulness course can significantly decrease perceived stress.
- The decrease in stress is maintained at 1 month follow-up and is comparable to other interventions.
- The online mindfulness course is an accessible and acceptable way for people to receive an intervention that can offer a way to decrease levels of perceived stress.

**Strengths and limitations of this study:**

- The sample consisted of people who had signed up to and paid for the online course that limits the extent to which we can extrapolate to those who might use it because referred by others (such as a health professional).
- The effect of practice on mindfulness was not examined, so it is unclear what mediates the change in perceived stress. Other factors contributing to a decrease in stress were not included in this preliminary evaluation.

Stress and worrying thoughts maintained over a prolonged period of time can have a number of negative effects on physical and mental health.^1–4^ There is a growing body of evidence demonstrating that a Mindfulness course, be that Mindfulness Based Stress Reduction (MBSR)^5^ ^6^ or Mindfulness Based Cognitive Therapy (MBCT),^7^ can be an effective intervention for a broad range of chronic health problems such as depression, chronic pain, anxiety disorders and stress and that they enhance the level of coping in everyday life.^8^ ^9^ Research also shows that perceived stress decreases after taking part in a Mindfulness intervention, and these benefits are maintained at follow-up between 1 and 3 months.^10–12^

As the awareness of the benefits of mindfulness therapy grows, so too does the need for access to this type of intervention. It has been noted that in the UK, the NHS cannot handle all of the need for mental health resources.^13^ One way to increase access to therapies that is becoming more popular is to create online courses. The benefit of any online therapy, in addition to the reduction in cost to the participant and health service, is the provision for the participant to do the therapy from their own home or other comfortable surroundings and in their own time. Yet perhaps the most important benefit of an effective online intervention is accessibility for a large number of people who may benefit from mindfulness and maybe unable to attend another course for various reasons.^14^ ^15^

There are reasons to believe that such an on-line approach might be appropriate: the results from several trials of online cognitive behavioural therapy for a range of disorders are promising, reporting reduced rates of depression relapse and appearing to be particularly helpful for prevention of future recurrence and reduction in antidepressant usage.^16^ ^17^ Similar results have been reported for Beating the Blues, with significant improvements for anxiety and depression, with increased cost-effectiveness than face-to-face Cognitive Behavioural Therapies.^18^

However, there are additional challenges for an on-line mindfulness course. This derives from the fact that mindfulness is normally taught in a group or class, and the developers of mindfulness-based interventions^6^ ^7^ suggest that the presence of others is an important part of the learning. Not only do they provide social support in the form of other participants who can share their experiences of symptoms and the meditation exercises, but participants learn much from the investigative dialogue between teacher and class participants after each mindfulness practice. It is therefore questionable whether on-line mindfulness teaching will prove useful at all.

In this study, therefore, we wished to investigate whether an online mindfulness course has a significant positive effect on the self-reported stress ratings of participants and whether the online course produces similar benefits as the mindfulness courses delivered face-to-face in groups as measured by the Perceived Stress Scale (PSS,^19^ ^20^ see online appendix A for a breakdown of previous research examining changes in PSS scores). Stress was decided on as the outcome measure, first, because we wanted to measure something that people could readily relate to. Second, a number of studies using mindfulness-based interventions have shown that they reduce stress and have used this measure, so we could ‘benchmark’ the impact of the online mindfulness course against the existing evidence-base. Third, we wished to examine whether the amount of home practice is related to any reduction in stress.

Our hypotheses are as follows:

1. Participants will report significantly lower PSS scores on completion of the online course in comparison to their pre-course PSS score.
2. The reduction in stress will be maintained at 1 month follow-up compared with their PSS scores taken before the course.
3. Participants who practice more throughout the course will have a larger decrease in their PSS scores; and
4. The decreases found in the PSS scores will be comparable to other face-to-face mindfulness interventions.

## Methods

### Participants

Study participants were self-referrals to an online mindfulness course. These participants were not recruited for this study specifically and consented for their data to be used anonymously for research upon registering. Participation in the online course was on a self-pay basis. Self-report data were collected prior to the start of the course, and there was an opportunity to give feedback 1 month after course completion.

### Procedure

The online intervention was a modified mindfulness course comprising elements of MBSR and MBCT. The online course costs £40 (∼US$60) and follows the same class sequence as the 8-week mindfulness course. The course is run by the Mental Health Foundation and Wellmind Media and was developed in conjunction with leading UK mindfulness instructors. The participants access instructional videos that guide the formal meditations, through the website (http://www.bemindfulonline.com).

The online course consists of 10 interactive sessions led by two mindfulness instructors, one male and one female. Participants learn to use formal meditation skills (body scan, mindful movement, sitting meditation, three minute breathing space) and informal mindfulness techniques (incorporating mindfulness into daily activities, such as mindful eating) through videos, assignments and emails. The course lasts for a minimum of 4 weeks, depending on when participants are able to complete the practice and homework logs. Participants are able to have a break from the course and receive email reminders to continue at the point that they last participated.

For each week, participants are asked to practice at least one formal exercise using the audio and video clips supplied, such as the body scan that lasts for 30 min, or mindful movement, lasting 10 min and one informal exercise in their own time, such as eating a meal mindfully. Home practice data are derived from online self-report questions enquiring how often the participant had been able to complete certain mindfulness activities during the week.

### Measures

The PSS^19^ is a widely used and validated scale that measures how much the individual perceives events as uncontrollable and overwhelming during the previous month. Validity and reliability of the PSS have been reported as good.^19^ Cronbach's α in this study was 0.72. The PSS consists of 10 items answered using five-point scales, each ranging from 0 to 4, with four being the highest stress score. The predictive validity is expected to change after 4–8 weeks because of the varying nature of life events and daily worries and their effect on perceived stress. The PSS has been used in previous research of mindfulness and has repeatedly shown a reduction in perceived stress scores using a mindfulness intervention.^10–12^ In this study, Perceived stress ratings were assessed before the intervention, immediately after completing the final practice log and at 1 month follow-up.

## Results

### Sample characteristics

Data from the first 100 participants who completed the course, including the 1-month follow-up, were analysed. The mean age of the participants was 48 years (SD=11.25, range 28–72) and 74% were women. The mean PSS score for the sample was higher than that provided as the norm distribution which is between 11.9 and 14.7.^20^ The average PSS score of this sample was comparable to previous samples of either ‘highly stressed’ individuals^21^ or individuals with a wide range of illness, personal or employment-related stress.^10^

The average time to finish the course was 6.14 weeks. Participants who did not report their practice were assumed not to have completed any meditation exercises for that week. A majority (90%) answered all the self-report practice questions; the rest answered at least eight questions out of 12 except one participant who completed only the first three, for which we assumed no practice for the remainder of the course. Seven of the 10 participants who did not finish all of their practice logs were men, but there were no significant differences in age, time taken to complete the course or PSS scores at any time point between those who practiced and those who did not.

### Changes in perceived stress

**Figure 1:**
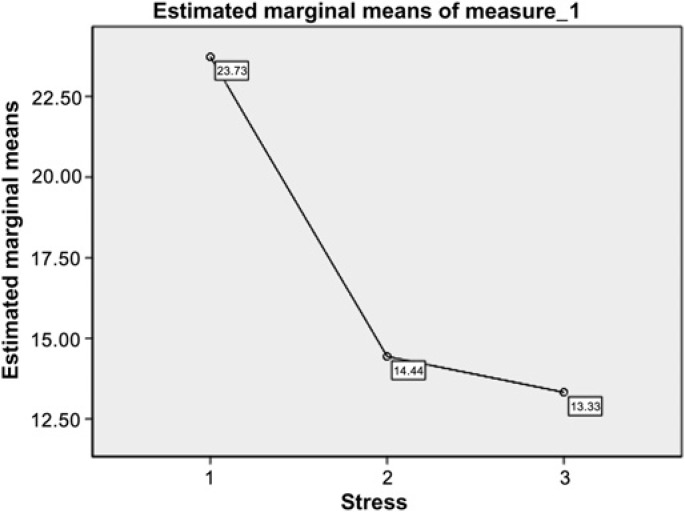
Mean PSS scores before and after course and at 1 month follow-up (N=100).

The changes in mean PSS before and after the course and after 1 month follow-up are shown in figure 1. The mean PSS score of the sample prior to the course was 23.73 (SD=9.95, range 10–38). The mean PSS score after the online mindfulness intervention was 14.44 (SD=5.86, range 1–30) and after 1 month was 13.30 (SD 6.40, range 0–29). Mean PSS score changes significantly from before to after the course (F (2, 98) = 138.7, p<0.001) and remained stable at 1 month follow-up. The pre-post effect size (d) was 1.57, comparable to other published studies of mindfulness courses in groups (see online appendix).

### Mindfulness practice

The sample was divided into three groups according to the amount of practice: high (‘every day or most days’ N=33), medium (‘sometimes’ N=55) and low (‘rarely’ N=12). There was no significant difference between the practice groups in their PSS score decrease; however, the group reporting the highest amount of practice had the highest stress score before the course (F (2, 97) = 143.4, p<0.001).

To investigate this trend further, the sample was split into two groups: people who practiced, on average, everyday or almost everyday (N=33) and people who practiced less (N=67). As before, the people who practiced more were more stressed to begin with (F (1, 98) = 203.3, p<0.001). Again, there was no significant difference between the practice groups in terms of their PSS score decrease after the course and at 1 month follow-up.

## Discussion

The aim of this preliminary study was to evaluate the feasibility of the online mindfulness course and to gather preliminary evidence on its efficacy on reducing perceived stress. This initial investigation suggests that people are able to use the course in this mode of delivery and are finding it helpful. As predicted, we found that participants' PSS scores significantly improved after completing the course and that their scores remain stable at 1 month follow-up. The hypothesis that participants who practiced more of the formal and informal mindfulness activities would experience a greater decrease in perceived stress was not supported but led us to find that participants who were more stressed at the outset practiced more and their PSS scores decreased to match the remainder of the sample at the 1-month follow-up. We found a trend in the expected direction for the amount of mindfulness practice improving PSS scores but this was not significant.

As this study is a preliminary investigation, before further discussion, it is important to take account of a number of limitations.

First, there was no control comparison, so we cannot be sure whether the online course was wholly responsible for the decline in perceived stress. There may have been other variables responsible, or the passage of time alone may result in such a decrease. This clearly needs to be addressed in future research. This is especially important as the group studied were people who were seeking the course and paying a small fee to undertake it. We do not know if it generalises to other populations, such as NHS patients in the UK, who do not pay for their treatment.

Second, even if the reduction in stress was not due to the enthusiasm of the people taking the course, we do not know which of many factors might have influenced the PSS scores. In particular, the effect of practice on trait mindfulness was not examined, so it is unclear even whether it is change in mindfulness that mediates the change in perceived stress. It is possible, for example, that stress is reduced by non-specific factors, such as participants feeling a higher sense of control over their own well being by taking part in the online programme. This aspect will be included in future research.

A third limitation is the way practice was self-reported by participants, so we cannot be sure how accurately practice was reported. Participants were not asked to state how many minutes they practiced certain exercises nor for how many days. Instead people self-reported how often they had completed a certain exercise over that week: ‘every day, most days, once or twice or never’. This may have been problematic for analysis as it may not accurately reflect how many times participants practiced different exercises; for example, we found that the course lasted, on average, 6 weeks instead of the expected 4 weeks because people are able to complete the weekly modules in their own time. As such, participants may not have been able to clearly represent how much they were practicing if the amount changed because of a break in the programme. It may be useful in future to ask how many days participants practiced each exercise and if the exercise is an informal one to ask how many times during that day they practiced, so that we get a clearer account of what participants did.

Fourth, we know little about the current sample and did not assess clinical status. In particular, anxiety and depressive symptoms were not assessed. In future research, it would be useful to see whether a group with clinical symptoms meeting ‘caseness’ criteria derive benefit, and to what extent, as well as continuing to investigate benefits to well-being in the wider population.

Finally, although only a trend was found in PSS score change depending on the amount of practice, we did find that people who were more stressed at the beginning practiced more formal and informal mindfulness practices. These participants then made a marked improvement, so that at 1 month follow-up, their PSS scores were similar to the rest of the sample. People who are feeling more stressed may be more motivated to work and therefore put more effort into learning the skills the online course has to offer than those who are less stressed. However, without a control group, it is not possible to rule out that this finding represents ‘regression to the mean’ of initial ‘outliers’. Future research should examine these possibilities as it will clarify whether this programme is useful even for participants who report persistent high levels of stress.

Bearing in mind these limitations, it is important to note that the changes in the PSS scores found in this study were comparable to other interventions (see online appendix A for a table outlining the change in PSS found in other studies), especially face-to-face MBSR and cognitive therapy courses that reported effects sizes *lower* than those found in this study (ranging from 0.52 to 1.19). However, our online course did not achieve an effect size as high as those found in psychopharmacological treatment research with clinical samples (ranging between 1.59 and 2.34), though we note that these samples start with much higher PSS scores prior to intervention.

This apparent comparability of the online course to other mindfulness and cognitive therapies delivered face-to-face is surprising: the online course seems to achieve similar results despite the fact that people are not part of a group and have no real relationship with a mindfulness teacher or other participants (often cited as critically important facilitators of change in class-based formats). It is possible that generalisation is helped by the fact that people learn the skills in the very same environment that they then use them, rather than taking time out of their everyday life to attend a mindfulness course in a different context.

It is not unexpected that the online course did not yield as significant a change in PSS as the psychopharmacological studies when considering the difference in samples. The research examining anti-depressants and their effect on perceived stress used clinical samples, usually people with Major Depressive Disorder, who had much higher PSS scores at the outset. One might expect a more significant decrease in PSS or an improvement in most facets of well being as participants had more scope for improvement.

Finally, we note that the teachers of this on-line course were experienced meditation practitioners, so we cannot infer from any degree of efficacy found with a shorter mindfulness-based courses such as this one, that *any* short course delivered by any therapist will thereby be effective.

In conclusion, the results from our preliminary investigation on the feasibility of an online mode for mindfulness-based intervention look promising. Given that the needs of the general public to find ways of reducing stress are enormous, research of such an accessible and cheap treatment intervention, so long as the quality and integrity is assured, can only be constructive to health services around the world and to those people who for whatever reason are unable to attend a class or therapy.
